# Can preoperative thyroglobulin antibody levels be used as a marker for well differentiated thyroid cancer?

**DOI:** 10.1186/s40463-016-0143-5

**Published:** 2016-05-14

**Authors:** S. Hosseini, R. J. Payne, F. Zawawi, A. Mlynarek, M. P. Hier, M. Tamilia, V. I. Forest

**Affiliations:** Faculty of Medicine, McGill University, Montreal, QC Canada; Department of Otolaryngology – Head and Neck Surgery, McGill University, Montreal, QC Canada; Department Otolaryngology - Head and Neck Surgery, King Abdulaziz University, Jeddah, Saudi Arabia; Division of Endocrinology, Jewish General Hospital, McGill University, Montreal, QC Canada

## Abstract

**Background:**

It has been reported that thyroglobulin antibody are more frequently elevated in patients with thyroid cancercompared to general population. This study aims at evaluating whether preoperative thyroglobulin antibody (TgAb) levels increase the likelihood that a thyroid nodule is malignant.

**Methods:**

A retrospective review of 586 patients who underwent thyroidectomy was conducted. Demographic data, TgAb levels, and final histopathology were recorded. Patients were divided into two groups: TgAb positive (defined as TgAb ≥ 30 IU/ml) and TgAb low/negative (defined as TgAb < 30).

**Results:**

Preoperative TgAb levels were available in 405 patients. There were 353 (87 %) patients in the TgAblow/negative group (malignancy rate: 50.42 %) and 52 (13 %) patients in the TgAb positive group (malignancy rate: 65.38 %). The sensitivity, specificity, positive predictive value and negative predictive value of TgAb ≥ 30 IU/ml for thyroid malignancy were 16.04 %, 90.67 %, 65.38 % and 49.58 %, respectively. The relative risk of having a malignant thyroid nodule when the TgAb titers were≥30 IU/ml was 1.30 (CI1.04-1.62) and the odds ratio was 1.86 (CI 1.01-3.41). Both the Pearson chi-square test (p = 0.024) and Fisher’s exact test (p = 0.017) yielded statistical significance between the two groups.

**Conclusions:**

In this study, patients with preoperative TgAb ≥ 30 IU/ml had a higher rate of malignancy when compared topatients with TgAb < 30 IU/ml. This suggests that an elevated TgAb level may indicate that a thyroid nodule is at an increased risk for malignancy.

## Background

Thyroid nodules are commonly encountered in the general population. It is estimated that there is a 5 to 10 % lifetime risk of having a thyroid nodule [1]. Among all thyroid nodules, about 5 % are cancerous independently of their size [2]. With the steady rise in the detection rate of thyroid nodules, there has been increased interest to identify parameters that can be used as risk factors and predictors of thyroid cancer [3–5].

As a matter of fact, in an attempt to identify other risk factors to help with risk stratification, serum thyroglobulin (Tg) and their antibodies have been studied. Previous reports have shown a relationship between elevated measurements of Tg and well-differentiated thyroid carcinoma (WDTC). Tg has been recognized as an established tumor marker for thyroid cancer [6–9]. However, serum thyroglobulin antibody (TgAb) levels may interact with Tg and give a lower serum Tg value. In fact, Tg complexed with TgAb cannot be detected by the currently available immunometric assay methods, which impairs in those cases the clinical utility of Tg as a prognostic factor for WDTC. However, this has lead to another question on the potential significance of TgAb in risk stratification of thyroid nodule.

Depending on the population studied and the assay used, TgAb levels are elevated in approximately 20 % of patients with WDTC, as compared to 10 % of individuals in the general population [10]. Also, high titers of TgAb are present in the serum of most patients with chronic lymphocytic thyroiditis (CLT). In 2010, Kim et al. reported for the first time that a positive TgAb test was an independent predictor of thyroid nodule malignancy, regardless of the presence of CLT [11]. Subsequently, other reports showed conflicting results. The aim of the present study is to assess whether higher levels of preoperative TgAb correlate with an increased likelihood of a thyroid nodule being malignant.

## Methods

This study is a retrospective review of 586 patients who underwent a thyroidectomy by a single surgeon at the McGill University teaching hospitals between January 2012 and December 2013. Our investigation obtained ethics approval by the McGill University Health Center Research Ethics Board and the Jewish General Hospital Research Ethics Office.

The inclusion criteria were age > 18 years old, hemi or total thyroidectomy, and available preoperative TgAb measurements and final histopathology reports. Patients with final diagnoses other than WDTC were excluded from the study. The histopathology analysis was done according to the World Health Organization Classification of Thyroid Tumors. CLT was identified on histopathological analysis. Data collated included patients’ demographic data, final histopathology reports, and preoperative TgAb measurements.

Patients were divided into two groups based on TgAb titers: TgAb positive group, defined as TgAb ≥30 IU/ml, and TgAb low/negative group, defined as TgAb <30 IU/mL. TgAb levels were measured using the Immulite 2000 anti-TgAb assays (Siemens, Llanberis, United Kingdom).

Patients with an incidental finding of micropapillary thyroid carcinoma without extrathyroidal extension (ETE) and/or lymph node (LN) positivity were recorded as having benign pathology. These carcinomas were categorized as such since they present an indolent behavior and a favorable prognosis, which suggest their resemblance to benign carcinomas [12]. Micropapillary carcinomas with unfavorable histopathological features, such as ETE and/or LN positivity, behave more aggressively and are hence more appropriately managed as malignant carcinomas [12].

Sensitivity, specificity, positive predictive value, negative predictive value, relative risk and odds ratio of TgAb ≥30 IU/ml as a diagnostic test for thyroid malignancy were calculated. The data was statistically analyzed using SPSS 20.0. Pearson chi-square and Fisher’s exact test were used to identify difference between groups. A *p* value of 0.05 or less was considered statistically significant.

## Results

### Clinical characteristics

Among the 586 patients who underwent thyroid surgery during the study period, 181 were excluded from the study: 174 had incomplete data (TgAb value or final histopathology not available), 6 had medullary carcinoma and 1 had anaplastic carcinoma. Among those with incomplete data, 157 patients had unrecorded TgAb values but available pathology reports, showing a rate of malignancy of 68.8 % (108/157) in this subgroup of patients. A total of 405 patients were included in this study. There were 329 females (81 %) and 76 (19 %) males, giving a female to male ratio of approximately 4:1. In the TgAb positive group, 92 % (48/52) of the patients were women in comparison to 80 % (282/353) in the TgAb negative group (*p* = 0.031). The mean age in the TgAb positive group was 45 years compared to 50 years in the TgAb negative group (*p* = 0.013).

**Fig. 1:**
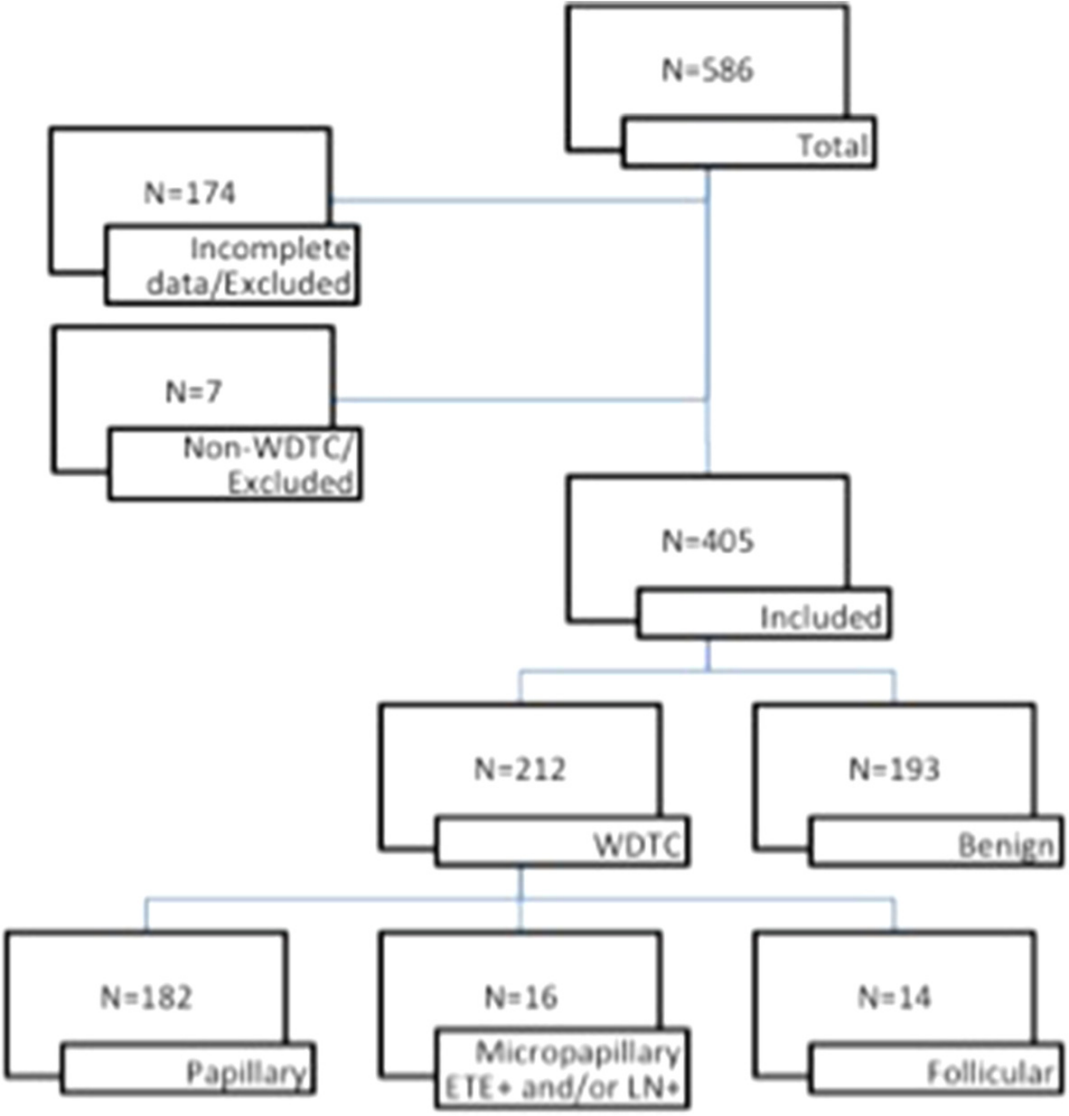
Flow chart of diagnosis

There were 212 (52 %) patients with WDTC: 182 (86 %) of which had papillary carcinoma, 16 (7 %) had micropapillary carcinoma with ETE and/or LN positivity, and 14 (7 %) had follicular carcinoma. There were 193 (48 %) patients with benign pathology, of which 68 (35 %) had micropapillary carcinoma without ETE and/or LN positivity. The flow chart of this study is shown in Fig. 1. The patient characteristics are presented in Table 1.

**Table 1:** Patient characteristics of the sample (*N* = 405)

	Benign	Malignant	*p*-value
	(*N* = 193)	(*N* = 212)	
Age (years)	49.53 ± 13.94	49.25 ± 13.87	NS
Gender, no. female (%)	164 (84.97)	165 (77.83)	NS
TgAb ≥ 30 IU/mL, no. (%)	18 (9.33)	34 (16.04)	0.024

Data represents mean ± standard deviation or number (percentage)

*NS* not significant

Among the 405 patients, 52 (13 %) were found to have TgAb levels ≥30 IU/mL, while the remaining 353 (87 %) had TgAb values <30 IU/mL. The TgAb values ranged from <20 (lowest value detected by the assay used) to 3362. A total of 340 (84 %) patients had values <20 while the median and mean TgAb values of the remaining patients were 96 and 412 (standard deviation 739). In the malignant group, 175 (83 %) patients had TgAb values <20 vs. 165 (85 %) in the benign group, while the median and mean TgAb values of the remaining patients were 108 vs. 53 and 751 (standard deviation 1920) vs. 313 (standard deviation 479) respectively.

**Fig. 2:**
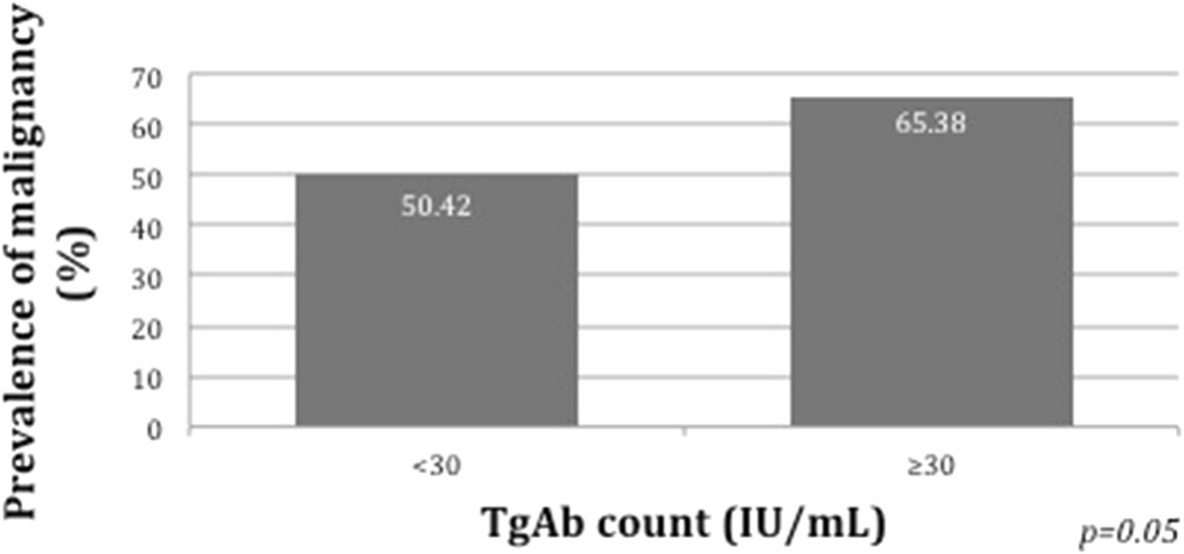
Prevalence of malignancy according to TgAb levels

There were 34 out of the 52 patients in the positive TgAb group who were diagnosed with WDTC, compared to 178 of the 353 patients in the low/negative TgAb group, resulting in a malignancy rate of 65 % vs. 50 %, respectively (*p* = 0.05). In other words, overall, 16 % of patients with WDTC had positive TgAb levels, compared to 9 % of the patients with a benign pathology (Table 1). The prevalence of malignancy according to TgAb levels is presented in Fig. 2. It is worth noting that within the benign group, 7.4 % of patients with micropapillary carcinoma without ETE and/or LN positivity had TgAb values ≥30 (5 out of 68) compared to 10.4 % (13 out of 125) of patients with other benign pathologies.

Overall, the final histopathology reports revealed the presence of CLT in 98 out of 405 patients (24.2 %). An approximately equal proportion of patients in both the malignant and benign groups presented with CLT (24.5 % vs. 23.8 % respectively). In the TgAb positive group, 50 % of patients with malignant disease had CLT. Similarly, 50 % of patients in the benign group harboured CLT in their thyroids. About 20 % of patients in the TgAb negative group had CLT, with once again an approximately equal proportion of patients in the malignant and benign groups (19.7 % vs. 21.1 % respectively). These results are shown in Table 2.

**Table 2:** Prevalence of specimens showing chronic lymphocytic thyroiditis according to TgAb group and final pathology

Number of specimens with CLT/total number of specimens (%)			
	Benign	Malignant	Total
TgAb ≥ 30	9/18 (50)	17/34 (50)	26/52 (50)
TgAb < 30	37/175 (21.1)	35/178 (19.7)	72/353 (20.4)
Total	46/193 (23.8)	52/212 (24.5)	98/405 (24.2)

*CLT* Chronic lymphocytic thyroiditis

### Statistical analysis

The sensitivity, specificity, positive predictive value and negative predictive value of TgAb ≥30 IU/ml as a predictive parameter of thyroid malignancy were 16.04 % (CI 11.37-21.68), 90.67 % (CI 85.66-94.38), 65.38 % (CI 50.91-78.03) and 49.58 % (CI 44.24-54.92), respectively. Both the Pearson chi-square test (*p* = 0.024) and Fisher’s exact test (*p* = 0.017) yielded statistical significance between the two groups.

## Discussion

This study demonstrates that the prevalence of WDTC was higher in patients with positive TgAb, compared to patients with low/negative TgAb. Our results suggest that a TgAb count ≥30 IU/ml may be specific for WDTC, although a lower count should not be used to rule out malignancy. In accordance with our data, other authors have reported that elevated TgAb levels could be an indicator that a thyroid nodule is at increased risk for malignancy. As mentioned earlier, Kim et al. were the first to report that a positive TgAb test was an independent predictor of thyroid nodule malignancy, regardless of the presence of CLT. A more recent study conducted by Grani et al. showed that an isolated TgAb positivity could be a mild risk factor for thyroid cancer, as opposed to Hashimoto’s thyroiditis, which did not correlate positively with malignant pathology [13].

Our study shows that in the TgAb positive group, there is a significantly higher proportion of women and patients were slightly younger compared to those in the TgAb negative group. Demographics and levels of TgAb were examined in the National Health and Nutrition Examination Survey (NHANES III), a large study that was conducted on the American population between 1988 and 1994 [14]. They reported that TgAb were more prevalent in women than in men. However, in this cohort, levels of TgAb were increasing with age when comparing youth vs adults vs elderly patients. We compared age according to the mean age of both groups which could explain the difference in this finding. The age groups were separated differently than our series of patients. Nevertheless, it is relevant to note that they lie in the same age category, as increasing concentrations with age in the NHANES III study was only significant when comparing groups with greater age difference (i.e. youth vs. adults vs. elderly).

Our results show that 50 % of TgAb positive patients had CLT compared to 20 % of TgAb negative patients. An association between the presence of TgAb and CLT has also been reported in the literature [15, 16]. However, we did not find a significant correlation between CLT and WDTC, as prevalence of CLT was the same for benign and malignant pathologies. This is in agreement with the literature. In fact, a population-based fine-needle aspiration biopsy study [17] and two large prospective studies with a 10-year follow-up [18, 19] did not find an association between CLT and WDTC. Currently, in the literature, there is uncertainty on whether CLT is a cause, a consequence or simply, histologically accompanying a malignancy, as both share some common pathways in molecular genetic pathology. It is also difficult to differentiate histologically between peritumoral lymphocytic infiltrations and true CLT [20–26]. However, most believe that pathologic processes of autoimmune thyroid disease have to be independent from the ones of tumorogenesis to explain the increased prevalence of CLT in TgAb positive patients.

In the aforementioned studies by Kim et al. [11] and Grani et al. [13], the patient’s final diagnostic outcome was established following FNAC, with only a minority of diagnoses confirmed by histological follow-up. One limitation of using FNAC as a diagnostic tool is that the cytological features observed in the sample obtained are not necessarily representative of the entire thyroid tissue and cannot consequently offer a definitive diagnosis. An alternative to this problem consists of using the final pathology report obtained after surgical excision, which is the only diagnostically conclusive method. However, the latter also presents limitations, namely the bias caused by the selection of patients with high suspicion of thyroid cancer, and hence requiring thyroidectomy. In order to balance this selection bias, our study focused on the comparison between a TgAb positive and a TgAb negative group. Moreover, selecting a sample of surgical candidates was especially relevant in the context where risk assessment algorithms are particularly needed for patients in whom surgery is considered a treatment option. It is worth noting that another source of potential selection bias stems from the relatively high malignancy rate (68.8 %) in patients excluded from the study due to unavailable TgAb values.

Finally, it is important to mention that in the current literature there is no defined threshold of what is considered an elevated TgAb titer in WDTC. Most studies published set their cut-off values according to the recommendations of the assay kit provided by the manufacturer, which are calculated for its use in the diagnosis of CLT. When used for that purpose, the cut-offs are set higher as it is believed that higher titers of TgAb are needed to interfere with Tg levels in patients with CLT compared to WDTC [27]. In fact, two studies suggested that up to 20 % of samples may be misclassified as TgAb negative when the manufacturer’s cut-off levels are used, as they are set too high [28, 29]. Since the manufacturer of the assay we used recommended a cut-off of 40 IU/mL, we lowered it to 30 IU/mL in an effort to capture more patients with potentially interfering TgAb values. This implies that a threshold of 30 IU/mL may not be transferable to centres using a different anti-TgAb assay with variable manufacturer’s recommendations. The reason for establishing the threshold to 30 and not to some other value below 40 relates to the statistical analysis of the test; a value of 30 offered the highest specificity among values showing statistical significance between the two groups.

Another limitation of our study is that it is retrospective. On one hand, this approach allowed us to accumulate data for a large number of cases; on the other hand, it limited our access to complete information for a number of patients. Nevertheless, we believe that the impact on the validity of our results is minimal as our results are concordant with other reports published in the literature. The results of the current study show that patients with elevated TgAb levels have a significantly higher rate of malignancy compared to patients with lower levels. However, this needs to be interpreted in the context of the aforementioned limitations. As such, incorporating TgAb levels measurement in the preoperative risk assessment of patients presenting with thyroid nodules could be helpful to predict malignancy when used with other variables. Nevertheless, an isolated TgAb test alone is not powerful enough and should not influence decision-making. Our results need confirmation from larger, prospective studies to better clarify the potential role of serum TgAb levels in the prediction of thyroid malignancy.

## Conclusions

Incorporating TgAb levels measurement in the preoperative risk assessment of patients presenting withthyroid nodules could be helpful to predict malignancy when used with other variables. Nevertheless, an isolated TgAb test alone is not powerful enough and should not influence decision-making. Our results need confirmation from larger, prospective studies to better clarify the potential role of serum TgAb levels in theprediction of thyroid malignancy.
